# Li-Fraumeni Syndrome: A Rare Genetic Disorder

**DOI:** 10.7759/cureus.29240

**Published:** 2022-09-16

**Authors:** Faria Tazin, Harendra Kumar, Muhammad A Israr, Deborah Omoleye, Vern Orlang

**Affiliations:** 1 Family Medicine, East Liverpool City Hospital, East Liverpool, USA; 2 Medicine, Dow University of Health Sciences, Karachi, PAK; 3 Department of Research, Larkin Community Hospital Palm Springs Campus, Hialeah, USA; 4 Division of Research, Grandville Medical Centre, Lagos, NGA

**Keywords:** family medicine, internal medicine, cancer, le fraumeni syndrome, hereditary diseases

## Abstract

Li-Fraumeni syndrome (LFS) is an inherited genetic condition that makes individuals predisposed to specific types of cancer. As a result, cancer risk can be passed down from generation to generation. TP53 is the genetic blueprint for a protein called p53 and most commonly causes this condition by mutations or alterations in that gene. Mutations prevent the gene from functioning properly. LFS is associated with TP53 gene mutations in approximately 70% of families. Most patients with LFS have one normal copy of TP53 and one mutated copy of TP53, usually inherited from a parent with the condition. This is a case report of a 40-year-old female who underwent genetic testing to determine her p53 mutation status. Her mother was diagnosed with breast cancer at a young age, despite the fact that her brothers and sisters' genetic tests came out normal. The genetic testing showed her as a carrier for the TP53 gene mutation. Despite the fact that she had no signs or symptoms of any linked tumors associated with the condition, she was diagnosed with LFS.

## Introduction

Certain individuals are at high risk of developing certain cancers. The most common types of cancer found in families with Li-Fraumeni syndrome (LFS) include osteosarcoma, acute leukemia, breast cancer, brain cancer, and adrenal cortical tumors [1]. An increased risk for melanoma, Wilms' tumor, stomach, colon, pancreas, esophagus, lung, and gonadal germ cell cancers has also been reported [1]. Recent studies have identified genetic events that modify the LFS phenotype. Several of these events are associated with the p53 pathway, including intragenic polymorphisms, mutations, polymorphisms, and aberrant copy number variations [2]. There is a 50% chance for a person to pass on the TP53 gene's normal copy to any child, regardless of whether they inherit a mutation or develop one de novo.

These genetic events partly explain why these LFS families may have broad tumor histotypes, accelerated onset ages, and different clinical outcomes. This case report presents a 40-year-old female patient who underwent genetic testing to determine her status. Her mother was diagnosed with breast cancer associated with the TP53 mutation at a very young age. Unfortunately, she tested positive for the p53 mutation. Although she did not develop cancer, she was on surveillance for the rest of her life.

## Case presentation

A 40-year-old woman visited the outpatient clinic and expressed concern about her mother's early breast cancer diagnosis. As her mother passed away prematurely due to breast cancer and did not go through any genetic testing, it was necessary to do a genetic test to determine her and her sibling's status for the TP53 mutation. She has been well, with no signs or symptoms of related cancers associated with the TP53 mutations, such as a history of a breast lump. There was no history of significant weight loss or weight gain. A complete physical examination was performed on her, as well as a thorough neurological examination, which revealed no additional information. Her vitals were normal. We requested total blood count, erythrocyte sedimentation rate, lactate dehydrogenase, testosterone, androstenedione, dehydroepiandrosterone sulfate, and abdomen and pelvic ultrasound normal. She underwent genetic counseling and testing that showed her as a carrier for the TP53 gene mutation. In Figure 1, the genetic test analyzed her for 42 genes known to cause hereditary breast, uterine, and colon cancer. The results showed a TP53 mutation. No mutation was detected in any of the other 41 genes. Her sibling's genetic tests were negative for the TP53 gene mutation. The patient was scheduled for prenatal testing, preimplantation genetic testing, annual whole-body and brain MRIs, as well as regular blood tests.

**Table 1:** The genetic test is positive for TP53 mutation

Genes	Mutation	Special notes
ATM, AXIN2, BARD1, BMPR1A, BRCA1, BRCA2, BRIP1, CDH1, CDKN2A, CHEK2, DICER1, EPCAM, GREM1, KIT, MEN1, MLH1, MSH2, MSH6, MUTYH, NBN, NF1, PALB2, PDGFRA, PMS2, POLD1, POLE, PTEN, RAD50, RAD51C, RAD51D, SDHA, SDHB, SDHC, SDHD, SMAD4, SMARCA4, STK11, TSC1, TSC2, VHL	No mutation was detected	Not applicable
TP53	Mutation was detected	c.542G>A

## Discussion

In 1969, Le Fraumeni syndrome was first identified and published as a rare hereditary cancer-prone condition with autosomal dominant inheritance [3]. Individuals with LFS have a significant chance of acquiring cancer, often young [4]. According to an epidemiologic study, males with LFS have a 73% lifetime risk of cancer, whereas women have a 100% lifetime risk [5]. Because TP53 mutations were identified as the fundamental cause, genetic testing for germline mutations in questionable families is now available [6,7]. The germline abnormalities linked with LFS are comparable to the somatic alterations observed in spontaneous cancers. Furthermore, patients with hereditary TP53 mutations are more prone to developing second primary tumors [2]. TP53 is a tumor suppressor gene. However, it is often altered via autosomal dominant inheritance, leaving patients with the wild-type allele, as seen in this example [8,9]. TP53 mutations in LFS families, on the other hand, have been demonstrated to be both heterozygous and homozygous [10].

Even though certain cancer-predisposition syndrome populations have the LFS pattern of inheritance and malignancy development, no TP53 mutations have been reported, emphasizing the involvement of other genes, notably MDM2 and CHEK2 [2]. An arginine was converted to a stop codon by a heterozygous autosomal dominant TP53 mutation at codon 306. There have only been 11 other cases with this similar mutational profile documented, one of which was hematologic cancer [11].

Secondary cancers are much more common in LFS patients with TP53 mutations than in those with normal TP53 [12]. Children may have a lengthier tumor growth time than adults due to their early onset. Once the TP53 gene mutation is present in a patient, the doctors should devise a clinical surveillance plan for early tumor diagnosis, cancer management, and treatment plan [13]. Clinical supervision has dramatically improved the five-year survival rate of LFS patients with TP53 mutations [14]. It is recommended that these young individuals get annual whole-body and brain MRIs. The complete blood count, erythrocyte sedimentation rate, lactate dehydrogenase, testosterone, androstenedione, and dehydroepiandrosterone sulfate are all markers that should be evaluated regularly using blood tests. In addition, abdomen and pelvic ultrasonography should be performed. A complete physical examination is also required, with specific attention paid to rapid weight and height increases, blood pressure, the growth curve, a Cushingoid look, male physical symptoms, and a thorough neurological evaluation. Once LFS is found, family members should be screened for associated genetics because they have a high chance of developing malignant neoplasms [15,16]. An appropriate and timely illness monitoring of TP53 mutations in LFS patients' family members from infancy may identify a variety of early medically diagnosed ailments, including breast cancer, soft tissue sarcoma, and other malignant neoplasms [17].

## Conclusions

LFS is a rare hereditary cancer-prone condition with autosomal dominant inheritance. People with LFS have an increased chance of developing cancer at a young age. Women have a 100% lifetime risk of acquiring cancer compared to a 73% incidence risk in males. Our patient is a 40-year-old female whose mother developed breast cancer early. She underwent genetic testing that showed her to be a carrier for the TP53 mutation, and she was confirmed to have LFS. Her siblings' genetic tests were negative for the TP53 mutation. The clinical surveillance performed on her for early tumor diagnosis showed no related signs and symptoms. Due to the increase in the five-year survival rate of LFS patients with TP53 mutations, it is paramount that these young individuals get whole-body and brain MRIs annually. Once LFS is confirmed, family members should be screened for associated genetics because they have a high chance of developing malignant neoplasms. To quickly identify associated cancers such as breast cancer, soft tissue sarcoma, and other malignant neoplasms, we urge adequate and rapid illness monitoring of TP53 mutations in the family members of LFS patients beginning in infancy.
